# Testicular Dysgenesis Syndrome and the Estrogen Hypothesis: A Quantitative Meta-Analysis

**DOI:** 10.1289/ehp.10545

**Published:** 2007-11-08

**Authors:** Olwenn V. Martin, Tassos Shialis, John N. Lester, Mark D. Scrimshaw, Alan R. Boobis, Nikolaos Voulvoulis

**Affiliations:** 1 Centre for Environmental Policy and; 2 Experimental Medicine and Toxicology Group, Imperial College London, United Kingdom; 3 Centre for Water Sciences, Cranfield University, Cranfield, United Kingdom; 4 Institute for the Environment, Brunel University, Uxbridge, United Kingdom

**Keywords:** cryptorchidism, diethylstilbestrol, endocrine disruption, environment, estrogen, hypospadias, meta-analysis, oral contraceptives, testicular cancer, testicular dysgenesis

## Abstract

**Background:**

Male reproductive tract abnormalities such as hypospadias and cryptorchidism, and testicular cancer have been proposed to comprise a common syndrome together with impaired spermatogenesis with a common etiology resulting from the disruption of gonadal development during fetal life, the testicular dysgenesis syndrome (TDS). The hypothesis that *in utero* exposure to estrogenic agents could induce these disorders was first proposed in 1993. The only quantitative summary estimate of the association between prenatal exposure to estrogenic agents and testicular cancer was published over 10 years ago, and other systematic reviews of the association between estrogenic compounds, other than the potent pharmaceutical estrogen diethylstilbestrol (DES), and TDS end points have remained inconclusive.

**Objectives:**

We conducted a quantitative meta-analysis of the association between the end points related to TDS and prenatal exposure to estrogenic agents. Inclusion in this analysis was based on mechanistic criteria, and the plausibility of an estrogen receptor (ER)-α–mediated mode of action was specifically explored.

**Results:**

We included in this meta-analysis eight studies investigating the etiology of hypospadias and/or cryptorchidism that had not been identified in previous systematic reviews. Four additional studies of pharmaceutical estrogens yielded a statistically significant updated summary estimate for testicular cancer.

**Conclusions:**

The doubling of the risk ratios for all three end points investigated after DES exposure is consistent with a shared etiology and the TDS hypothesis but does not constitute evidence of an estrogenic mode of action. Results of the subset analyses point to the existence of unidentified sources of heterogeneity between studies or within the study population.

Impaired spermatogenesis, male reproductive tract abnormalities such as hypospadias and cryptorchidism, and testicular cancer have been proposed to comprise a common underlying syndrome with a common aetiology resulting from the disruption of embryonic programming and gonadal development during fetal life, termed the testicular dysgenesis syndrome (TDS) (Sharpe and Skakkebaek 2003; Skakkebaek et al. 2001). A hormonal etiology most likely underlies this syndrome, although it is believed to have more than one cause, possibly including other than endocrine disruption. Some common causes of endocrine disruption include infection, diet and body weight, lifestyle, genetics, and environmental exposure, but endocrine-disrupting chemicals (EDCs), particularly those with estrogen-like properties, have received the most scientific attention.

The synthetic estrogenic drug diethylstilbestrol (DES) was prescribed to more than 5 million pregnant women from the late 1940s to the early 1970s to prevent abortions and pregnancy-related complications (Palmlund et al. 1993). Evidence later showed that maternal ingestion of DES during early pregnancy increased the risk of vaginal clear cell adenocarcinoma in female offspring (Herbst et al. 1971) and resulted in an increased incidence of malformations of the testes, the development of epididymal cysts, and impaired sperm quality in male offspring (Bibbo et al. 1977). During pregnancy, maternal estrogen levels are significantly elevated. However, more than 90% of maternal endogenous estrogens are effectively sequestered via binding to sex hormone binding globulin (SHBG), and thus the fetus is relatively protected (Joffe 2001; Vidaeff and Sever 2005). On the other hand, DES and ethinylestradiol do not bind well to SHBG, having a higher biopotency if ingested (Sharpe and Skakkebaek 2003; Vidaeff and Sever 2005). Additionally, transgenerational exposure is also possible when lipophilic xenoestrogens are mobilized during pregnancy and lactation (Colborn et al. 1993).

Previous systematic reviews of studies in which pregnant women were exposed to estrogens other than DES have failed to find evidence of an increased risk of urogenital abnormalities in the male offspring (Raman-Wilms et al. 1995; Storgaard et al. 2006; Toppari et al. 1996; Vidaeff and Sever 2005), and have raised the possibility that nonestrogenic or atypical estrogenic effects of DES exposure *in utero* induce male reproductive abnormalities. However, none of the effects of DES exposure on either male or female offspring of exposed wild-type pregnant mice were induced when administered to ERKO (ER-α knockout) mice (Couse et al. 2001), strongly suggesting an ER-α–mediated mechanism. There is, however, a body of experimental data that is consistent with an effect of antiandrogenic industrial chemicals on male sexual differentiation (Gray et al. 1999, 2000). Moreover, mechanisms other than endocrine disruption may be involved in testicular toxicity; for example, the nematocide dibromochloropropane, an alkylating agent, is one of the most potent known testicular toxins in adults (Joffe 2001). In this review we focus on the estrogen hypothesis of TDS.

Although several systematic reviews of the literature on the association between estrogenic agents and the disorders thought to belong to the TDS have been published, they are predominantly qualitative and the only quantitative summary estimate of the association between prenatal exposure to estrogenic agents and testicular cancer was published over 10 years ago (Toppari et al. 1996). The primary objective of a quantitative meta-analysis is to combine the results of previous studies examining a specific research question to arrive at a summary conclusion about a body of research. It has been found particularly useful when individual studies are too small to yield a valid conclusion, but it cannot, however, correct for bias and confounding. When applied to observational studies, subset analysis can be a useful tool to explore the reasons for discrepancies among the results of different studies.

The objectives of this research were therefore to carry out a quantitative meta-analysis of the association between three of the end points related to TDS and prenatal exposure to estrogenic agents that would account for both the size and quality of the studies included and yield updated summary estimates in light of the body of research carried out since the formulation of the estrogen hypothesis. Inclusion in this analysis was based on mechanistic criteria, and the plausibility of an ER-α–mediated mode of action was specifically explored. Moreover, subset analysis has been applied to categories of compounds with estrogenic potencies differing by several orders of magnitude in an attempt to detect the existence of any potency–response trend. Most of the studies of sperm quantity or quality have been concerned with time trends rather than etiology, and this end point was not considered further here.

## Material and Methods

### Identification and selection of literature

A computerized search was conducted using the databases PubMed (National Center for Biotechnology Information 2007) and Web of Science (ISI Web of Knowledge 2007) for the period 1970 to April 2007. The general search keywords were “estrogen,” “risk,” “dose,” and either “hypospadias,” “cryptorchidism,” or “testicular cancer.” A preliminary identification was performed by screening the titles and, if relevant, the abstracts of retrieved literature. The next stage was to check the citations and references of selected studies. This was an iterative process, repeated until no new study could be identified. A set of both inclusion and exclusion criteria was defined, and all relevant literature was then checked for eligibility. The inclusion criteria considered were *a*) study design, namely, either a case–control, cohort, or clinical trial; *b*) written in English; *c*) exposure to one or a mixture of known estrogenic compounds; and *d*) sufficient data reported to be used in meta-analysis.

The following exclusion criteria were used:

- Exposure to a group of compounds (suspected endocrine disruptors) for which mode of action was unspecified, for example, pesticides.
- Studies of exposure to phytoestrogens. Some phytoestrogens have been found to have a greater binding affinity for ER-β than for ER-α and can result in agonistic or antagonistic effects (Mueller et al. 2004).
- Studies of maternal endogenous hormones.
- Studies of the same cohort as this would bias the results towards the particular studies.
- Incomplete data.

### Data extraction and quality rating

In addition to the number of exposed and nonexposed cases and controls, and risk ratios (RRs) with their confidence intervals (CIs), information regarding the study design, estrogenic agent, geographic location of the study, and year of publication were extracted from the selected literature to allow subset analysis to be carried out. When more than one RR was reported, the following priorities were set for choice:

- Adjusted RRs were used, except when the study provided only unadjusted estimates.
- When multiple estimates were given, the RR estimator on which the authors had relied for their assessment of causal association was used.
- Overall RRs were chosen instead of those derived from further stratifications. If an overall estimate was not provided, the RRs of the maximum duration of exposure or the maximum exposure concentration were chosen.

Several aspects of the quality of each study were also recorded according to a rating scheme adapted from those previously described (Altman 1991; Rushton 2000). Every criterion was assessed on a scale of 0 to 2, 0 suggesting that it was not present, 1 when it was unclear, and 2 when that criterion was satisfied. A maximum score of 50 and 52 could be assigned for retrospective (case–control) and prospective (cohort and clinical trials) studies, respectively. This enabled a quality sensitivity analysis to be performed to check the influence of studies with low quality on the pooled estimate.

### Data analysis

#### Graphical representation

The RRs and CIs were plotted against the year of publication to determine whether any positive or negative trends in reporting RRs had occurred over time. Similarly, quality scores were plotted against the year of publication to investigate whether the quality of studies improved over time. To assess publication bias, a funnel plot (SE vs. RR) was produced based on the assumption that smaller studies are less precise in their RRs and thus have less weight and larger SE and should scatter more widely at the lower end of the graph, whereas larger studies will tend to be closer together (Sterne et al. 2001). Forest plots present the RRs against the reference of the study and help check homogeneity visually.

#### Statistical pooling

Pooled estimates and 95% CIs were calculated using both a fixed-effects model (Mantel–Haenszel method) and a random-effects model (DerSimonian–Laird method), allowing evaluation of the dependence of the conclusions of the analysis on the model assumptions. A summary estimate is considered statistically significant at the 0.05 level if its CI does not include unity.

The Mantel–Haenszel pooled effect estimate was used in a chi-square statistical test of homogeneity to assess the between-study variance. The magnitude of the test statistics depends on the weight of each study. When the number of studies is low or the studies themselves are small, the test statistic *Q* tends to be small. Tests of heterogeneity in meta-analyses are generally low in their power to reject the null hypothesis of homogeneity. For this reason, the chi-square statistical test of homogeneity was carried out at both 0.05 and 0.1 significance levels. Additionally, pooled estimates calculated using fixed effect and random effect models differ only if there is lack of homogeneity between studies. The estimates obtained by both methods were therefore compared to better assess potential heterogeneity between studies, in which case a single summary estimate of effect may be considered inappropriate.

#### Subset and sensitivity analyses

To investigate potential sources of heterogeneity between studies, we performed subset analyses for the study design, estrogenic agent, and geographic location.

Some studies exploring the influence of hormonal treatment during pregnancy did not specify the type of hormone. From what is known of the hormonal treatment of common conditions occurring during pregnancy, it was deemed reasonable to assume that they would have been likely to include estrogens, and these studies were included in the analysis. The validity of this assumption was tested by applying stricter criteria and calculating a summary estimate of effect excluding any study in which the hormone used had not been specified. Further sensitivity analysis was performed by excluding low-quality studies and extremes (exclusion of the studies with the largest and smallest RR estimators and exclusion of the studies with the largest and smallest weights) to verify that either the quality of the studies or one particular study did not have an excessive influence on the pooled estimate.

## Results

A total of 50 studies were identified for the association between *in utero* exposure to estrogenic agents and hypospadias and/or cryptorchidism, including 16 that had not been included in previous systematic reviews. Sixteen studies, of which 8 were new studies, were included in the calculation of a summary estimate of effect for either or both end points (Table 1). Studies predating the formulation of the TDS hypothesis often were designed to explore the association of *in utero* exposure to a range of pharmaceuticals with birth malformations. Other than 2 recent studies for which pesticide exposure was determined by chemical analysis of specific compounds, assessment of exposure to pesticides is generally derived from the occupation of the mother and specific agents are not identified.

Of the 12 studies identified for the association with testicular cancer, only 3 were excluded from the calculation of a summary estimate of effect (Table 2).

### Hypospadias

**Figure 1:**
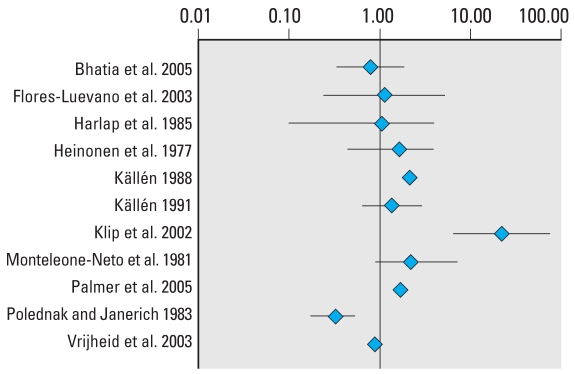
Forest plot of the risk estimates and their 95% CIs from the studies included in the meta-analysis of the association between prenatal exposure to estrogenic agents and hypospadias.

**Figure 2:**
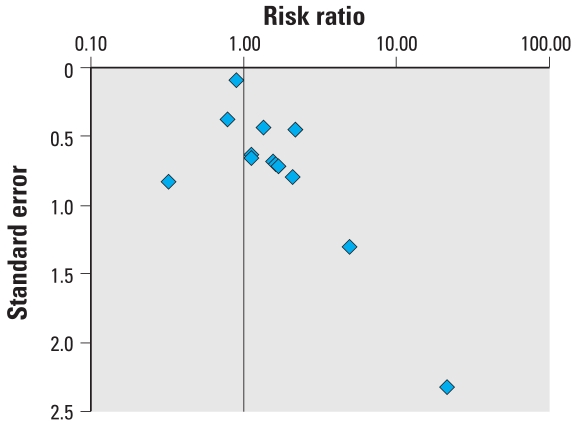
Funnel plot of the risk estimate studies included in the meta-analysis of the association between prenatal exposure to estrogenic agents and hypospadias and their SEs.

The data from studies included in the meta-analysis for hypospadias are summarized in Table 3. Three extreme values, two greater than and one lower than unity, can be identified visually from the forest plot of the RRs and their CIs (Figure 1). These extremes correspond to studies with larger SEs, and the shape given to the funnel plot (Figure 2) by those smaller positive studies would be consistent with publication bias. These two extreme positive risk ratios were, however, reported after what is commonly referred to as “third-generation exposure” to DES, when the mother herself had been exposed to DES prenatally. It was recognized that the inclusion of such studies in the meta-analysis could have introduced heterogeneity, and the influence of this choice was investigated in the subset analysis. Plots of the quality score and RRs versus year of publication did not suggest any significant trends in quality of the studies or estimates of effect over time (not shown).

The pooled estimates of effect by both the Mantel–Haenszel and DerSimonian–Laird methods are very close to unity, and no relationship between *in utero* exposure to estrogenic agents and hypospadias could be detected (Table 4). None of the chi-square tests allowed the rejection of the null hypothesis of homogeneity between the studies at the 0.05 or 0.1 level of statistical significance. The subsets of studies in which exposure to DES and pharmaceutical estrogens were investigated yielded statistically significant risk ratios with both models, although the modest discrepancy between the fixed-effects and random-effects estimates suggests heterogeneity. Summary estimates for the latter subset were no longer significant when studies that included DES exposure were excluded. Although these results were based on four studies that all addressed *in utero* exposure to oral contraceptives, some heterogeneity between studies remained. Excluding the studies of third-generation exposure to DES, values for the summary estimate of effect were found to be 1.33 (95% CI, 0.63–2.83) by the Mantel–Haenszel method and 1.31 (95% CI, 0.52–3.26) by the DerSimonian–Laird method, a very modest and nonsignificant increase in risk. Excluding third-generation exposure from the DES subset yielded estimates of 2.02 (95% CI, 1.12–3.65) by the Mantel–Haenszel method and 2.00 (95% CI, 0.97–4.15) by the DerSimonian–Laird method, on the basis of two studies investigating exposure to any estrogenic drug during the first trimester of pregnancy. The difference between the results obtained by the two models for studies of third-generation exposure to DES was reduced only slightly by excluding the study by Klip et al. (2002); the Mantel–Haenszel method yielded an estimate of 2.46 (95% CI, 0.91–6.67) and the DerSimonian–Laird method of 2.18 (95% CI, 0.64–7.46). The latter study’s cohort had been recruited in a fertility clinic, and whether results obtained with subfertile women are generalizable to all women exposed to DES *in utero* has been questioned (Hernandez-Diaz 2002).

Although the equality of the results obtained by both methods for the environmental estrogens subset suggests those results are robust, the influence of the weight of the study by Vrijheid et al. (2003) cannot be underestimated, as shown by the sensitivity analysis. Exclusion of this study from the analysis yielded a statistically significant Mantel–Haenszel estimate but a lower and not statistically significant DerSimonian–Laird estimate, revealing heterogeneity. A statistically significant estimate was obtained for prospective studies by the Mantel–Haenszel method, but the wide difference with the estimate using the random effect model was suggestive of heterogeneity. Geographic subsets point to a higher risk in Latin America, although the pooled estimates for this location were based on only two studies and did not reach statistical significance.

In addition to the results of the sensitivity analysis presented in Table 4, a pooled estimate of effect was calculated when a stricter inclusion criterion was applied, namely, excluding results from the study by Monteleone-Neto et al. (1981). This had little influence on the overall result, generating summary estimates of 0.97 (95% CI, 0.83–1.13) for the fixed effect model or 0.93 (95% CI, 0.80–1.09) for the random effect model.

### Cryptorchidism

**Figure 3:**
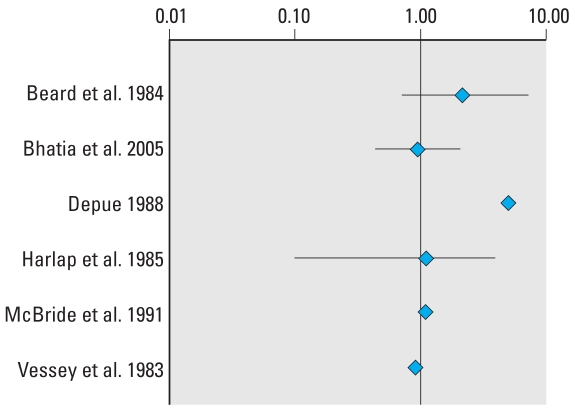
Forest plot risk estimates and their 95% CIs from the studies included in the meta-analysis of the association between prenatal exposure to estrogenic agents and cryptorchidism.

**Figure 4:**
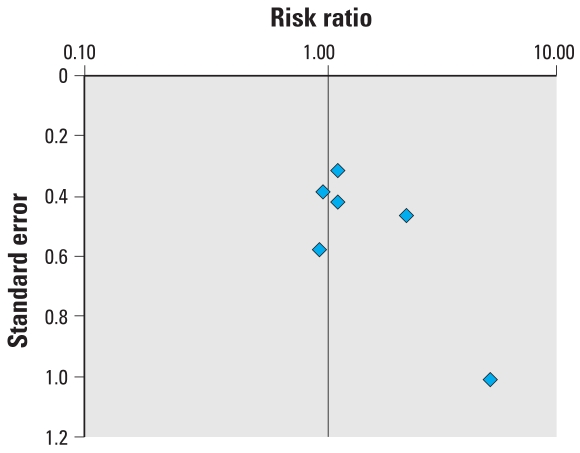
Funnel plot of the risk estimate studies included in the meta-analysis of the association between prenatal exposure to estrogenic agents and cryptorchidism and their SEs.

Data for the six studies included in the meta-analysis for cryptorchidism can be found in Table 5. The results of only two studies significantly differ from unity, as illustrated by the forest plot (Figure 3). The small number of eligible studies renders analysis of the funnel plot and potential for publication bias difficult (Figure 4). The SEs do, however, illustrate well that the studies were all relatively small. No time trends for the estimate of effect or the quality of studies could be detected (not shown).

As presented in Table 6, the pooled estimates of effect by both the Mantel–Haenszel and DerSimonian–Laird methods are marginally superior to unity, and their relative divergence implies there may be sources of heterogeneity. Chi-square tests did not, however, detect that any of the subsets were significantly heterogeneous. Excluding studies in which DES exposure was examined, either exclusively or along with hormonal therapeutics, yielded summary estimates consistent with no relationship. Statistical pooling of the studies including DES exposure generated a statistically significant estimate by the Mantel–Haenszel method, suggesting a doubling of the risk of cryptorchidism after *in utero* exposure to DES. The same estimate by the DerSimonian–Laird method did not, however, reach statistical significance and the difference relative to the fixed effect model is indicative of heterogeneity. The heterogeneity introduced by the DES subset of studies can again be observed by comparing the results obtained for all pharmaceutical estrogens with those obtained by pooling the two studies of accidental use of oral contraceptives during pregnancy. Study design also appeared to be a source of heterogeneity. If case–control studies are prone to recall bias, this subset also included the study with the highest estimate, itself a source of heterogeneity, as shown by the sensitivity analysis. Excluding the study by Depue (1988) reduced the difference between estimates by both models, the Mantel–Haenszel estimate then calculated as 1.29 (95% CI, 0.87–1.91) and that by the DerSimonian–Laird method as 1.23 (95% CI, 0.81–1.86). This was also observed for the American subset of studies. When the Depue (1988) study is omitted, the Mantel–Haenszel method yielded a no longer statistically significant estimate of 1.34 (95% CI, 0.84–2.14) and the DerSimonian–Laird method an estimate of 1.27 (95% CI, 0.72–2.23).

Applying a stricter exclusion criterion to studies examining hormonal treatment did not affect which studies were included in the meta-analysis of cryptorchidism. The study with the highest weight appears to lower the overall estimates, whereas increasing quality seems to reduce heterogeneity and lower the estimate of effect toward unity. These variations did not, however, influence the overall conclusion that aside from the DES studies subset, summary estimates did not detect any association between *in utero* exposure to estrogenic substances and cryptorchidism.

### Testicular cancer

**Figure 5:**
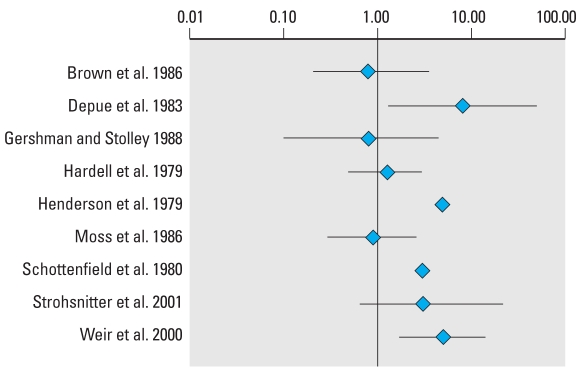
Forest plot risk estimates and their 95% CIs from the studies included in the meta-analysis of the association between prenatal exposure to estrogenic agents and testicular cancer.

**Figure 6:**
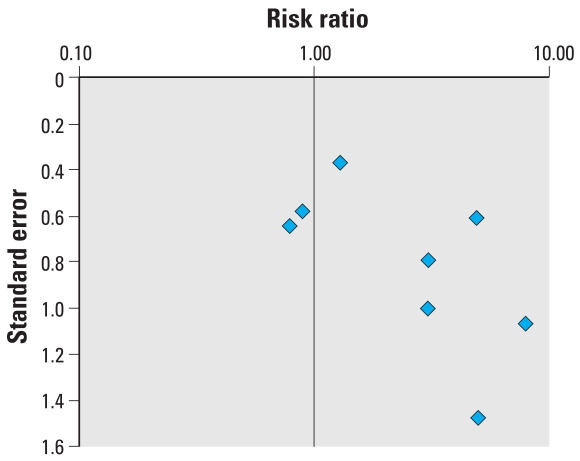
Funnel plot of the risk estimate studies included in the meta-analysis of the association between prenatal exposure to estrogenic agents and testicular cancer and their SEs.

**Figure 7:**
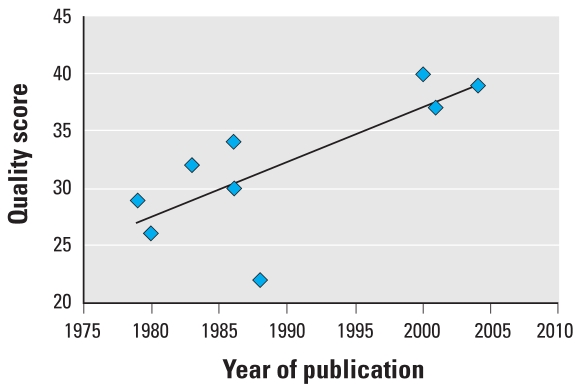
Time trend for quality showing quality score attributed to studies included in the meta-analysis of the association between prenatal exposure to estrogenic agents and testicular cancer by year of publication. *R*^2^ = 0.5711.

Nine studies were included in the meta-analysis of testicular cancer and the data used are summarized in Table 7. Of these, 4 had not been included in the summary estimate previously calculated by Toppari et al. (1996). The lack of homogeneity between studies is evident from the forest plot (Figure 5). Further, the funnel plot (Figure 6) also illustrates the relatively small size of the included studies. Although a positive trend over time was found for the quality of the included studies (Figure 7), no significant time trend could be detected for the effect size (not shown).

Both the fixed and random effect models yield a statistically significant estimate; however, the discrepancy between the two results is suggestive of heterogeneity despite the result from the chi-square test (Table 8). Conversely, the subset analysis was limited by the similarity of the question addressed by the studies included. Eight of the nine studies were interested in hormonal exposure and were conducted in the United States. Despite this, statistically significant heterogeneity between the studies was detected at the 0.1 level. Pooling the two studies examining DES exposure specifically produced a raised but statistically nonsignificant result. Despite the unexplained heterogeneity, all estimates that were calculated point to a doubling of the risk of developing testicular cancer after exposure to estrogenic agents *in utero*. The work on chlorinated biphenyls (PCBs) by Hardell et al. (2004) was the only study examining environmental estrogens. Its size was relatively small, and it did not detect such an effect.

Applying a stricter exclusion criterion to studies examining hormonal treatment excluded four studies from the meta-analysis; namely, Brown et al. (1986), Gershman and Stolley (1988), Henderson et al. (1979), and Weir et al. (2000). This resulted in a slightly lower Mantel–Haenszel estimate of 1.98 (95% CI, 1.23–3.18) and if the DerSimonian–Laird estimate remained equal to 1.59, because of the wider confidence interval (95% CI, 0.93–2.69), statistical significance was no longer achieved. The sensitivity analysis is consistent with some heterogeneity between the studies, the estimates obtained being relatively sensitive to the exclusion of particular studies varying above and below a risk estimate of 2. The quality of the studies seemed to explain at least some of this heterogeneity.

## Discussion

While it is clear that hypospadias, cryptorchidism, and testicular cancer are all positively associated with prenatal exposure to DES, this meta-analysis was unable to produce evidence that such effects were associated with environmental estrogens or even accidental use of oral contraceptives during pregnancy. This is consistent with the results obtained in earlier meta-analyses (Raman-Wilms et al. 1995; Toppari et al. 1996).

The main limitations of meta-analysis are *a*) the susceptibility of its summary results to publication bias, *b*) the influence of the quality of studies, *c*) the possibility of including multiple results from the same study, and finally, *d*) heterogeneity between studies that could lead to invalid conclusions. The methodology employed in this present review attempts to address these issues. Additionally, the importance of carrying out and reporting a sensitivity analysis was illustrated by the case of hypospadias where the weight attributed to one particularly large study had a nonnegligible influence on the results. In this particular case, the study by Vrijheid et al. (2003) inferred exposure to phthalates from registry data about occupation, and although such an approach can allow the analysis of a great number of cases, assessment of exposure is much more likely to be prone to confounding. The number of studies included in meta-analyses lies typically between 5 and 15, and the results presented here also fall within this range. The size of the homogeneity test statistic depends on both the number and size of individual studies. The funnel plots offer a good visual representation of the precision and size of individual studies, and it is clear that most studies published on the association between estrogenic agents and the probable end points of a TDS were found to be relatively small. The chi-square tests had, therefore, a relatively low power to detect heterogeneity. However, in the absence of statistical heterogeneity, the results of the fixed effect and random effect models should be virtually identical, and the comparison of results obtained by applying both the Mantel–Haenszel and DerSimonian–Laird models enabled the exploration of sources of heterogeneity despite this low statistical power.

If the quality of the studies was found to explain some of the heterogeneity observed, particularly in the case of testicular cancer, the remaining heterogeneity could not be explained solely by the fact that environmental, and therefore generally much weaker, estrogens were included in the analysis. The systematic review of published literature yielded relatively few studies examining the association of male urogenital abnormalities or testicular cancer with environmental estrogens specifically; a number of studies concerned with an association with broad categories of putative endocrine disruptor, most often pesticides, were excluded from the meta-analyses. This illustrates the difficulties associated with assessment of exposure, pesticide exposure often being inferred from parental occupation rather than direct measurement. Furthermore, there is increasing evidence that, in accordance with pharmacokinetic theory, the effects of xenobiotics acting via the same mechanism can be predicted fairly accurately by concentration addition (Zhu et al. 2006). Accurately accounting for combined exposure or adjusting for the confounding introduced by environmental exposures will probably require the development of mechanism-specific biomarkers of exposure.

When DES is excluded, there is no conclusive evidence of an effect of pharmaceutical estrogens. Exposure to such estrogens is related mainly to the accidental use of oral contraceptives during pregnancy or hormonal pregnancy tests. Such estrogenic pharmaceuticals often are given in combination with progestagens, and it is legitimate to question whether unopposed estrogens would have the same effects as opposed estrogens. This also highlights another difficulty associated with exposure assessment, that of critically sensitive periods of development and the ascertainment of whether exposure took place during a “window” of susceptibility to hormone disruption. Nonetheless, studies in which maternal levels of hormones were measured in the first and third trimester of pregnancy do not support an association with elevated estrogen levels but rather indicate that a lower estrogen/androgen ratio and/or higher levels of α-fetoproteins may be beneficial (McGlynn et al. 2005; Zhang et al. 2005). If in animals both estrogenic and antiandrogenic compounds have been associated with end points consistent with those of human TDS (Fisher 2004; Veeramachaneni 2000), epidemiologic evidence remains elusive. Alternatively, the doubling of the risk estimates of all three effects associated with DES exposure would be consistent with a shared etiology and the TDS hypothesis. It does not constitute conclusive evidence of an estrogenic mode of action, however, as common etiologic factors could be related to the underlying condition for which DES was prescribed. Furthermore hypospadias, cryptorchidism, and testicular cancer have all been found to be associated with low birth weight, suggesting a potential association with an underlying placental defect.

The understanding of the importance of endogenous estrogens in normal adult testicular function is becoming clearer. Their roles during fetal life, however, remain relatively unclear, but those mediated by the ER-αor ER-β have been shown to differ (Habert et al. 2006). Interestingly, DES has been found to have similar affinity for both receptors, whereas estradiol has only a slightly stronger affinity for ER-αcompared with ER-β (Mueller et al. 2004). ER-α has been detected in undifferentiated gonads as early as 10 days postconception in the mouse and found to be localized in the Leydig cells of fetal testis in rodents (Habert et al. 2006). Studies of the expression of ER-α and ER-β in human and nonhuman primates have so far yielded inconsistent results. Gaskell et al. (2003) reported that ER-α could not be detected in human fetal testes between weeks 12–19 of gestation, whereas Shapiro et al. (2005) found that ER-α was apparent by week 12, its levels peaked at 16 weeks before diminishing, and it was localized in Leydig cells. Current research focus has shifted to the role played by testosterone, anti-Müllerian hormone and insulin-like factor 3 produced by the fetal testes during masculinization. In the male rat, exposure to high levels of estrogens has been shown not only to suppress testosterone production but also to downregulate the expression of the androgen receptor protein in reproductive target tissues including the testes, Wolffian duct, and prostate (Sharpe 2006). Further research in this area may help shed light on possible mechanisms of injury or relevance of the rodent model.

The subset analyses did not generate many clues to explain the heterogeneity of the collected data. This is, however, consistent with the wide geographic variability in the incidence of the conditions of interest (Boisen et al. 2004; Richiardi et al. 2004). Interactions between genetic susceptibility and the environment have been the focus of research in this area (Martin et al. 2007), and advances in genomics have allowed the identification of polymorphisms associated with hypospadias, cryptorchidism, and testicular cancer (Beleza-Meireles et al. 2006; Kurahashi et al. 2005; Starr et al. 2005; Yoshida et al. 2005). Such discoveries may, however, give rise to as many questions as they offer to answer. This is well illustrated by the recent identification of the association of a variant of the gene for the ER-α with hypospadias and cryptorchidism in Japanese cohorts (Watanabe et al. 2007; Yoshida et al. 2005) that has now been found to be associated with a decreased incidence of hypospadias in a European cohort (Galan et al. 2007).

## Conclusion

The modest increase in risk for all three end points associated with DES exposure is consistent with a shared etiology and the TDS hypothesis, whereas the results of the subset analyses suggest the existence of yet unidentified sources of heterogeneity between studies or within the study populations. Although 10 years of further research on the potential effects of endocrine disruptors on male reproductive health have provided some clues regarding the etiology and mechanism of conditions such as hypospadias, cryptorchidism, and testicular cancer, there is still no conclusive evidence of the role played by environmental estrogens.

## Figures and Tables

**Table 1:** Studies identified for the association between *in utero* exposure to estrogenic agent and hypospadias and cryptorchidism.

Reference	End point	Comment	Previous reviews^a^
Aarskog 1970	Hypospadias	Data on progestins treatment only	
Beard et al. 1984	Cryptorchidism	Included	R-W, T, S
Beral and Colwell 1981	Cryptorchidism	Study too small to calculate risk ratio	Sx
Berkowitz and Lapinski 1996	Cryptorchidism	Use of clomiphene before pregnancy recognized	
Bernstein et al. 1988	Cryptorchidism	Maternal endogenous hormones	S
Bhatia et al. 2005	Cryptorchidism	Included	
	Hypospadias	Included	
Bianca et al. 2003	Hypospadias	Occupational exposure of fathers to pesticides	
Burton et al. 1987	Cryptorchidism	Maternal endogenous hormone levels	S
Calzolari et al. 1986	Hypospadias	Oral contraceptive use before pregnancy recognized	R-Wx, S
Cosgrove et al. 1977	Cryptorchidism Hypospadias	No control data for documented abnormalities	R-Wx, Sx
Czeizel et al. 1979	Hypospadias	Progesterone treatment	R-Wx
Czeizel et al. 1999	Cryptorchidism Hypospadias	Ecological study design	
Davies et al. 1986	Cryptorchidism	Oral contraceptive use before pregnancy recognized	S
Depue 1984	Cryptorchidism	Same cohort as Depue (1988)	R-Wx, S
Depue 1988	Cryptorchidism	Included	
Flores-Luevano et al. 2003	Hypospadias	Included	
Garcia-Rodriguez et al. 1996	Cryptorchidism	Ecological study design	V, S
Gill et al. 1977	Cryptorchidism Hypospadias	No genitourinary abnormalities in exposed infants	T
Gill et al. 1979	Cryptorchidism	Cryptorchidism in men with testicular hypoplasia	T, S
Harlap et al. 1975	Cryptorchidism Hypospadias	All cases exposed to progesterone	R-W
Harlap and Eldor 1980	Cryptorchidism Hypospadias	No cases after oral contraceptive use during pregnancy	R-W
Harlap et al. 1985	Cryptorchidism	Included	R-W
	Hypospadias	Included	
Heinonen et al. 1977	Hypospadias	Included	
Hemminki et al. 1999	Hypospadias	No exposed controls	
Henderson et al. 1976	Cryptorchidism	No unexposed cases	T, V
	Hypospadias	No unexposed cases	
Janerich et al. 1980	Hypospadias	Data for hypospadias not reported	R-Wx
Källén 1988	Hypospadias	Included	R-W
Källén and Winberg 1982	Hypospadias	No exposed controls	R-Wx, S
Källén et al. 1991	Hypospadias	Included	R-W, S
Key et al. 1996	Cryptorchidism	No exogenous hormone use	S
Klip et al. 2002	Hypospadias	Included	
Kristensen et al. 1997	Cryptorchidism	Exposure to unspecified pesticides	V, S
Longnecker et al. 2002	Cryptorchidism Hypospadias	DDE is antiandrogenic	V, S
McBride et al. 1991	Cryptorchidism	Included	R-W, S
Monteleone-Neto et al. 1981	Hypospadias	Included	R-W, V, S
North and Golding 2000	Hypospadias	Phytoestrogens	S
Palmer et al. 2005	Hypospadias	Included	
Pierik et al. 2004	Cryptorchidism Hypospadias	Phytoestrogens, unspecified pesticides, or EDCs	
Polednak and Janerich 1983	Hypospadias	Included	R-W, S
Pons et al. 2005	Hypospadias	Included	
Restrepo et al. 1990	Cryptorchidism	Unspecified pesticides	V
Rothman and Louik 1978	Hypospadias	Oral contraceptive use before pregnancy recognized	S
Sorensen et al. 2005	Hypospadias	Clomiphene is estrogenic but does not act via ER	
Stoll et al. 1990	Hypospadias	Oral contraceptive use before pregnancy recognized	R-Wx, S
Sweet et al. 1974	Hypospadias	No exogenous estrogens during pregnancy	R-W
Torfs et al. 1981	Cryptorchidism Hypospadias	Same cohort as Bhatia et al. (2005)	R-Wx
Vessey et al. 1983	Cryptorchidism	Included	S
	Hypospadias	No unexposed cases	S
Vrijheid et al. 2003	Hypospadias	Included	
Weidner et al. 1998	Cryptorchidism Hypospadias	Exposure to unspecified pesticides	V, S
Whitehead and Leiter 1981	Cryptorchidism	No controls	T

a The letters R-W, T, V, and S refer to Raman-Wilms et al. (1995), Toppari et al. (1996), Vidaeff and Sever (2005), and Storgaard et al. (2006), respectively, where the suffix “x” indicates study was excluded from that review.

**Table 2:** Studies identified for the association between *in utero* exposure to estrogenic agent and testicular cancer.

Reference	Comment	Previous reviews^a^
Brown et al. 1986	Included	T, S
Depue et al. 1983	Included	T, S
Dieckmann et al. 2001	Maternal endogenous hormone levels	
Gershman and Stolley 1988	Included	S
Hardell et al. 2004	Included	
Hemminki et al. 1999	No cases	
Henderson et al. 1979	Included	T, S
Moss et al. 1986	Included	T, S
Schottenfeld et al. 1980	Included	T, S
Strohsnitter et al. 2001	Included	S
Walcott et al. 2002	Phytoestrogens	
Weir et al. 2000	Included	S

a The letters T and S stand for Toppari et al. (1994) and Storgaard et al. (2006), respectively.

**Table 3:** Summary of data used for the meta-analysis of the association between prenatal estrogenic agents and hypospadias.

				Cases		Controls					
Reference	Design	Agent	Location	E	NE	E	NE	RR (95% CI)	SE	Weight	Quality score
Bhatia et al. 2005	Case–control	DDT	California, USA	9	34	42	117	0.79 (0.33–1.89)	0.38	7.07	41
Flores-Luevano et al. 2003	Case–control	DDT	Mexico City	8	33	5	23	1.13 (0.24–5.29)	0.65	2.39	37
Harlap et al. 1985	Cohort	Oral contraceptives	North Carolina, USA	3	98	847	32,597	1.10 (0.10–3.90)	0.64	2.47	36
Heinonen et al. 1977	Cohort	Estrogenic drugs	United States	4	184	295	25,069	1.60 (0.44–4.04)	0.69	2.12	45
Källén et al. 1988	Case–control	Oral contraceptives	Sweden	5	43	6	109	2.11	0.79	1.58	23
Källén et al. 1991	Case–control	Oral contraceptives	8 countries	16	830	11	835	1.36 (0.64–2.92)	0.43	5.40	30
Klip et al. 2002	Cohort	DES (mother exposed prenatally)	Netherlands	4	8	205	8,729	21.30 (6.50–70.10)	2.34	0.18	27
Monteleone-Neto et al. 1981	Case–control	Sex hormones	Latin America	21	252	12	307	2.20 (1.04–4.91)	0.44	5.11	24
Palmer et al. 2005	Cohort	DES (mother exposed prenatally)	United States	10	3	2,522	1,336	1.70 (0.40–6.80)	0.72	1.95	36
Polednak and Janerich 1983	Case–control	Oral contraceptives	New York, USA	1	98	3	96	0.33	0.82	1.48	27
Pons et al. 2005	Case–control	DES (mother exposed prenatally)	Paris, France	3	44	237	17,349	4.99 (1.20–16.80)	1.30	0.59	17
Vrijheid et al. 2003	Case–control	Phthalates (occupational)	United Kingdom	147	3,324	1,399	31,092	0.90 (0.74–1.10)	0.09	129.31	31

Abbreviations: E, exposed; NE, nonexposed.

**Table 4:** RRs (95% CIs) of the summary estimate of effect, subsets, and sensitivity analyses for the association between hypospadias and prenatal exposure to estrogenic agents.

Subset of studies	No. of studies included	Mantel–Haenszel method (fixed effects)	χ^2^*p*-Value	DerSimonian–Laird method (random effects)
All studies	12	1.02 (0.88–1.19)	0.30	1.16 (0.83–1.62)
Excluding DES exposure	7	0.93 (0.79–1.09)	0.69	0.91 (0.78–1.07)
Studies including DES exposure	5	2.49 (1.54–4.02)	0.75	2.14 (1.15–3.98)
Mothers exposed to DES prenatally	3	3.73 (1.58–8.80)	0.40	2.54 (0.78–8.33)
Pharmaceutical estrogens only	9	1.85 (1.30–2.64)	0.45	1.54 (1.00–2.36)
Pharmaceutical estrogens excluding DES	4	1.27 (0.74–2.19)	0.36	1.13 (0.61–2.10)
Environmental estrogens only	3	0.90 (0.76–1.06)	0.89	0.90 (0.76–1.06)
European studies	4	0.96 (0.81–1.14)	0.18	0.96 (0.72–1.27)
North American studies	5	1.03 (0.63–1.68)	0.50	0.93 (0.56–1.55)
Latin American studies	2	1.86 (0.99–3.48)	0.39	1.78 (0.87–3.64)
Excluding highest risk ratio	11	1.00 (0.86–1.16)	0.37	0.99 (0.82–1.20)
Excluding lowest risk ratio	11	1.03 (0.87–1.20)	0.35	1.02 (0.84–1.25)
Excluding highest weight	11	1.55 (1.13–2.11)	0.42	1.29 (0.90–1.85)
Excluding lowest weight	11	1.00 (0.86–1.16)	0.37	0.99 (0.82–1.20)
Case–control studies only	8	0.98 (0.84–1.15)	0.22	1.00 (0.78–1.28)
Cohort studies only	4	2.10 (1.14–3.85)	0.54	1.46 (0.59–3.57)
Excluding studies with quality score < 30	7	0.94 (0.80–1.10)	0.85	0.93 (0.79–1.09)
Excluding studies with quality score < 35	5	1.11 (0.69–1.77)	0.83	1.06 (0.65–1.73)

**Table 5:** Summary of data used for the meta-analysis of the association between prenatal estrogenic agents and cryptorchidism.

				Cases		Controls					
References	Design	Agent	Location	E	NE	E	NE	RR (95% CI)	SE	Weight	Quality score
Beard et al. 1984	Case–control	Estrogenic drugs	Minnesota, USA	9	104	15	211	2.20 (0.70–7.20)	0.47	4.60	34
Bhatia et al. 2005	Case–control	DDT	California, USA	11	32	42	117	0.95 (0.43–2.07)	0.39	6.65	41
Depue 1988	Case–control	Estrogenic drugs	United States	5	380	3	765	5.15	1.01	0.99	29
Harlap et al. 1985	Cohort	Oral contraceptives	North Carolina, USA	6	196	844	27,595	1.10 (0.10–3.90)	0.42	5.78	36
McBride et al. 1991	Case–control	Oral contraceptives	British Columbia, Canada	18	226	34	454	1.10	0.31	10.50	38
Vessey et al. 1983	Clinical trial	DES	United Kingdom	6	6	138	126	0.91	0.58	3.00	18

Abbreviations: E, exposed; NE, nonexposed.

**Table 6:** RRs and 95% CIs of the summary estimate, subsets and sensitivity analyses for the association between cryptorchidism and prenatal exposure to estrogenic agents.

Subset of studies	No. of studies included	Mantel–Haenszel method (fixed effects)	χ^2^*p*-Value	DerSimonian–Laird method (random effects)
All studies	6	1.34 (0.96–1.87)	0.44	1.22 (0.86–1.73)
Excluding DES exposure	3	1.06 (0.70–1.59)	0.95	1.05 (0.70–1.59)
Studies including DES	3	2.09 (1.13–3.86)	0.24	1.80 (0.83–3.93)
Pharmaceutical estrogens	5	1.44 (0.99–2.10)	0.37	1.31 (0.87–1.96)
Pharmaceutical extrogens excluding DES	2	1.10 (0.49–2.49)	1	1.10 (0.49–2.49)
Case–control studies	4	1.45 (0.98–2.15)	0.24	1.38 (0.81–2.34)
Cohort studies	2	1.04 (0.53–2.02)	0.79	1.03 (0.53–2.00)
American studies	4	1.55 (1.00–2.39)	0.24	1.40 (0.82–2.41)
Excluding highest risk ratio	5	1.21 (0.86–1.72)	0.66	1.16 (0.81–1.66)
Excluding lowest risk ratio	5	1.38 (0.97–1.97)	0.34	1.27 (0.86–1.87)
Excluding highest weight	5	1.46 (0.97–2.19)	0.32	1.30 (0.82–2.06)
Excluding lowest weight	5	1.21 (0.86–1.72)	0.66	1.16 (0.81–1.66)
Excluding studies with quality score < 30	4	1.25 (0.86–1.80)	0.53	1.19 (0.82–1.73)
Excluding studies with quality score < 35	3	1.06 (0.70–1.59)	0.95	1.05 (0.70–1.59)

**Table 7:** Summary of data used for the meta-analysis of the association between prenatal estrogenic agents and testicular cancer.

				Cases		Controls					
Reference	Design	Agent	Location	E	NE	E	NE	RR (95% CI)	SE	Weight	Quality score
Brown et al. 1986	Case–control	Sex hormones	Washington, DC, USA	4	198	5	201	0.80 (0.20–3.50)	0.64	2.43	30
Depue et al. 1983	Case–control	Estrogenic drugs	Los Angeles, USA	8	88	2	103	8.00 (1.30–49)	1.07	0.88	32
Gershman and Stolley 1988	Case–control	DES	Connecticut, USA	4	75	5	74	0.80 (0.10–4.50)	0.65	2.37	22
Hardell et al. 2004	Case–control	Estrogenic PCBs	Sweden	29	29	30	31	1.30 (0.50–3.00)	0.37	7.31	39
Henderson et al. 1979	Case–control	Hormone treatment	Los Angeles, USA	6	72	1	77	5.00	1.47	0.46	29
Moss et al. 1986	Case–control	DES or other hormones	California and Nevada, USA	7	202	6	204	0.90 (0.30–2.60)	0.59	2.89	34
Schottenfeld et al. 1980	Case–control	DES or other hormones	United States	11	170	3	133	3.05	0.79	1.61	26
Strohsnitter et al. 2001	Cohort	DES	United States	6	2	1,359	1,392	3.05 (0.65–21.96)	1.01	0.99	37
Weir et al. 2000	Case control	Hormone treatment	Ontario, Canada	15	310	7	483	4.90 (1.70–13.90)	0.61	2.66	40

Abbreviations: E, exposed; NE, nonexposed.

**Table 8:** RRs and 95% CIs of the summary estimates, subsets and sensitivity analyses for the association between testicular cancer and prenatal exposure to estrogenic agents.

	No. of studies included	Mantel–Haenszel method (fixed effects)	χ^2^*p*-Value	DerSimonian–Laird method (random effects)
All studies	9	2.14 (1.48–3.10)	0.12	1.59 (1.04–2.43)
DES exposure exclusively	2	2.53 (0.79–8.09)	0.77	2.47 (0.61–10.00)
Pharmaceutical estrogens	8	2.57 (1.66–3.99)	0.09	1.94 (0.98–3.87)
Case–control studies only	8	2.10 (1.43–3.07)	0.09	1.71 (0.92–3.17)
North American studies	8	2.57 (1.66–3.99)	0.09	1.94 (0.98–3.87)
Excluding highest risk ratio	8	1.89 (1.29–2.78)	0.21	1.56 (0.93–2.61)
Excluding lowest risk ratio	8	2.31 (1.56–3.40)	0.14	1.94 (1.08–3.48)
Excluding highest weight	8	2.57 (1.66–3.99)	0.09	1.94 (0.98–3.87)
Excluding lowest weight	8	2.08 (1.42–3.03)	0.10	1.68 (0.95–2.97)
Excluding studies with quality score < 30	6	2.16 (1.42–3.29)	0.08	1.79 (0.91–3.52)
Excluding studies with quality score < 35	3	2.33 (1.39–3.91)	0.13	2.23 (0.98–5.05)

## References

[b1-ehp0116-000149] Aarskog D (1970). Clinical and cytogenetic studies in hypospadias. Acta Paediatr Scand.

[b2-ehp0116-000149] Altman DG (1991). Practical Statistics for Medical Research.

[b3-ehp0116-000149] Beard CM, Melton LJ, Ofallon WM, Noller KL, Benson RC (1984). Cryptorchism and maternal estrogen exposure. Am J Epidemiol.

[b4-ehp0116-000149] Beleza-Meireles A, Omrani D, Kockum I, Frisen L, Lagerstedt K, Nordenskjold A (2006). Polymorphisms of estrogen receptor beta gene are associated with hypospadias. J Endocrinol Invest.

[b5-ehp0116-000149] Beral V, Colwell L (1981). Randomised trial of high doses of stilboestrol and ethisterone therapy in pregnancy: long-term follow-up of the children. J Epidemiol Commun H.

[b6-ehp0116-000149] Berkowitz GS, Lapinski RH (1996). Risk factors for cryptorchidism: a nested case-control study. Paediatr Perinat Epidemiol.

[b7-ehp0116-000149] Bernstein L, Pike MC, Depue RH, Ross RK, Moore JW, Henderson BE (1988). Maternal hormone levels in early gestation of cryptorchid males—a case-control study. Br J Cancer.

[b8-ehp0116-000149] Bhatia R, Shiau R, Petreas M, Weintraub JM, Farhang L, Eskenazi B (2005). Organochlorine pesticides and male genital anomalies in the child health and development studies. Environ Health Perspect.

[b9-ehp0116-000149] Bianca S, Li Volti G, Caruso-Nicoletti M, Ettore G, Barone P, Lupo L (2003). Elevated incidence of hypospadias in two Sicilian towns where exposure to industrial and agricultural pollutants is high. Reprod Toxicol.

[b10-ehp0116-000149] Bibbo M, Gill WB, Azizi F, Blough R, Fang VS, Rosenfield RL (1977). Follow-up study of male and female offspring of DES-exposed mothers. Obstet Gynecol.

[b11-ehp0116-000149] Boisen KA, Kaleva M, Main KM, Virtanen HE, Haavisto AM, Schmidt M (2004). Difference in prevalence of congenital cryptorchidism in infants between two Nordic countries. Lancet.

[b12-ehp0116-000149] Brown LM, Pottern LM, Hoover RN (1986). Prenatal and perinatal risk factors for testicular cancer. Cancer Res.

[b13-ehp0116-000149] Burton MH, Davies TW, Raggatt PR (1987). Undescended testis and hormone levels in early-pregnancy. J Epidemiol Commun H.

[b14-ehp0116-000149] Calzolari E, Contierro MR, Roncarati E, Mattiuz PL, Volpato S (1986). Aetiological factors in hypospadias. J Med Genet.

[b15-ehp0116-000149] Colborn T, vom Saal FS, Soto AM (1993). Developmental effects of endocrine-disrupting chemicals in wildlife and humans. Environ Health Perspect.

[b16-ehp0116-000149] Cosgrove MD, Benton B, Henderson BE (1977). Male genitourinary abnormalities and maternal diethylstilbestrol. J Urol.

[b17-ehp0116-000149] Couse JF, Dixon D, Yates M, Moore AB, Ma L, Maas R (2001). Estrogen receptor-[alpha] knockout mice exhibit resistance to the developmental effects of neonatal diethylstilbestrol exposure on the female reproductive tract. Dev Biol.

[b18-ehp0116-000149] Czeizel A, Toth J, Erodi E (1979). Aetiological studies of hypospadias in Hungary. Hum Hered.

[b19-ehp0116-000149] Czeizel AE, Hegedus S, Timar L (1999). Congenital abnormalities and indicators of germinal mutations in the vicinity of an acrylonitrile producing factory. Mutat Res.

[b20-ehp0116-000149] Davies TW, Williams DRR, Whitaker RH (1986). Risk-factors for undescended testis. Int J Epidemiol.

[b21-ehp0116-000149] Depue RH (1984). Maternal and gestational factors affecting the risk of cryptorchidism and inguinal hernia. Int J Epidemiol.

[b22-ehp0116-000149] Depue RH (1988). Cryptorchidism, an epidemiologic-study with emphasis on the relationship to central nervous-system dysfunction. Teratology.

[b23-ehp0116-000149] Depue RH, Pike MC, Henderson BE (1983). Estrogen exposure during gestation and risk of testicular cancer. J Natl Cancer I.

[b24-ehp0116-000149] Dieckmann KP, Endsin G, Pichlmeier U (2001). How valid is the prenatal estrogen excess hypothesis of testicular germ cell cancer?. Eur Urol.

[b25-ehp0116-000149] Fisher JS (2004). Environmental antiandrogens and male reproductive health: focus on phthalates and testicular dysgenesis syndrome. Reproduction.

[b26-ehp0116-000149] Flores-Luevano S, Farias P, Hernandez M, Romano-Riquer P, Weber JP, Dewailly E (2003). DDT/DDE concentrations and risk of hypospadias. A case-control pilot study. Salud Publica Mexico.

[b27-ehp0116-000149] Galan JJ, Guarducci E, Nuti F, Gonzalez A, Ruiz M, Ruiz A (2007). Molecular analysis of estrogen receptor alpha gene AGATA haplotype and SNP12 in European populations: potential protective effect for cryptorchidism and lack of association with male infertility. Hum Reprod.

[b28-ehp0116-000149] Garcia-Rodriguez J, Garcia-Martin M, Nogueras-Ocana M, de Dios Luna-del-Castillo J, Espigares Garcia M, Olea N (1996). Exposure to pesticides and cryptorchidism: geographical evidence of a possible association. Environ Health Perspect.

[b29-ehp0116-000149] Gaskell TL, Robinson LLL, Groome NP, Anderson RA, Saunders PTK (2003). Differential expression of two estrogen receptor beta isoforms in the human fetal testis during the second trimester of pregnancy. J Clin Endocrinol Metabol.

[b30-ehp0116-000149] Gershman ST, Stolley PD (1988). A case-control study of testicular cancer using Connecticut tumour registry data. Int J Epidemiol.

[b31-ehp0116-000149] Gill WB, Schumacher GFB, Bibbo M (1977). Pathological semen and anatomical abnormalities of the genital tract in human male subjects exposed to diethylstilbestrol *in utero*. J Urol.

[b32-ehp0116-000149] Gill WB, Schumacher GFB, Bibbo M, Straus FH, Schoenberg H, Schoenberg HW (1979). Association of diethylstilbestrol exposure in utero with cryptorchidism, testicular hypoplasia and semen abnormalities. J Urology.

[b33-ehp0116-000149] Gray LE, Ostby J, Furr J, Price M, Veeramachaneni DNR, Parks L (2000). Perinatal exposure to the phthalates DEHP, BBP, and DINP, but not DEP, DMP, or DOTP, alters sexual differentiation of the male rat. Toxicol Sci.

[b34-ehp0116-000149] Gray LE, Ostby J, Monosson E, Kelce WR (1999). Environmental antiandrogens: low doses of the fungicide vinclozolin alter sexual differentiation of the male rat. Toxicol Ind Health.

[b35-ehp0116-000149] Habert R, Delbes G, Duquenne C, Livera G, Levacher C (2006). Effets des estrogènes sur le développement du testicule pendant la vie foétale et néonatale [in French]. Gynécol Obstét Fert.

[b36-ehp0116-000149] Hardell L, Malmqvist N, Ohlson CG, Westberg H, Eriksson M (2004). Testicular cancer and occupational exposure to polyvinyl chloride plastics: a case-control study. Int J Cancer.

[b37-ehp0116-000149] Harlap S, Eldor J (1980). Births following oral contraceptive failures. Obstet Gynecol.

[b38-ehp0116-000149] Harlap S, Prywes R, Davies AM (1975). Birth defects and oestrogens and progesterones in pregnancy [Letter]. Lancet.

[b39-ehp0116-000149] Harlap S, Shiono PH, Ramcharan S (1985). Congenital abnormalities in the offspring of women who used oral and other contraceptives around the time of conception. Int J Fertil.

[b40-ehp0116-000149] Heinonen OP, Slone D, Shapiro S (1977). Birth Defects and Drugs in Pregnancy.

[b41-ehp0116-000149] Hemminki E, Gissler M, Toukomaa H (1999). Exposure to female hormone drugs during pregnancy: effect on malformation and cancer. Br J Cancer.

[b42-ehp0116-000149] Hemminki K (2004). Familial risk in testicular cancer as a clue to a heritable and environmental aetiology. Br J Cancer.

[b43-ehp0116-000149] Henderson BE, Benton B, Cosgrove M, Baptista J, Aldrich J, Townsend D (1976). Urogenital tract abnormalities in sons of women treated with diethylstilbestrol. Pediatrics.

[b44-ehp0116-000149] Henderson BE, Benton B, Jing J, Yu MC, Pike MC (1979). Risk-factors for cancer of the testis in young men. Int J Cancer.

[b45-ehp0116-000149] Herbst AL, Ulfelder H, Poskanzer DC (1971). Adenocarcinoma of the vagina. N Engl J Med.

[b46-ehp0116-000149] Hernandez-Diaz S (2002). Iatrogenic legacy from diethylstilbestrol exposure. Lancet.

[b47-ehp0116-000149] ISI Web of Knowledge (2007). ISI Web of Knowledge Home Page.

[b48-ehp0116-000149] Janerich DT, Piper JM, Glebatis DM (1980). Oral-contraceptives and birth-defects. Am J Epidemiol.

[b49-ehp0116-000149] Joffe M (2001). Are problems with male reproductive health caused by endocrine disruption?. Occup Environ Med.

[b50-ehp0116-000149] Källén B (1988). Case-control study of hypospadias, based on registry information. Teratology.

[b51-ehp0116-000149] Källén B, Mastroiacovo P, Lancaster PAL, Mutchinick O, Kringelbach M, Martinezfrias ML (1991). Oral-contraceptives in the etiology of isolated hypospadias. Contraception.

[b52-ehp0116-000149] Källén B, Winberg J (1982). An epidemiological study of hypospadias in Sweden. Acta Paediatr Scand.

[b53-ehp0116-000149] Key TJA, Bull D, Ansell P, Brett AR, Clark GMG, Moore JW (1996). A case-control study of cryptorchidism and maternal hormone concentrations in early pregnancy. Br J Cancer.

[b54-ehp0116-000149] Klip H, Verloop J, van Gool JD, Koster ME, Burger CW, van Leeuwen FE (2002). Hypospadias in sons of women exposed to diethylstilbestrol in utero: a cohort study. Lancet.

[b55-ehp0116-000149] Kristensen F, Irgens LM, Andersen A, Bye AS, Sundheim L (1997). Birth defects among offspring of Norwegian farmers, 1967–1991. Epidemiology.

[b56-ehp0116-000149] Kurahashi N, Sata F, Kasai S, Shibata T, Moriya K, Yamada H (2005). Maternal genetic polymorphisms in CYP1A1, GSTM1 and GSTT1 and the risk of hypospadias. Mol Hum Reprod.

[b57-ehp0116-000149] Longnecker MP, Klebanoff MA, Brock JW, Zhou HB, Gray KA, Needham LL (2002). Maternal serum level of 1,1-dichloro-2,2-bis(*p*-chlorophenyl)ethylene and risk of cryptorchidism, hypospadias, and polythelia among male offspring. Am J Epidemiol.

[b58-ehp0116-000149] Martin OV, Lester JN, Voulvoulis N, Boobis AR (2007). Human health and endocrine disruption: a simple multicriteria framework for the qualitative assessment of end point-specific risks in a context of scientific uncertainty. Toxicol Sci.

[b59-ehp0116-000149] McBride ML, Vandensteen N, Lamb CW, Gallagher RP (1991). Maternal and gestational factors in cryptorchidism. Int J Epidemiol.

[b60-ehp0116-000149] McGlynn KA, Graubard BI, Nam JM, Stanczyk FZ, Longnecker MP, Klebanoff MA (2005). Maternal hormone levels and risk of cryptorchism among populations at high and low risk of testicular germ cell tumors. Cancer Epidemiol Biomarkers Prev.

[b61-ehp0116-000149] Monteleone-Neto R, Castilla EE, Paz JE (1981). Hypospadias—an epidemiological-study in Latin-America. Am J Med Genet.

[b62-ehp0116-000149] Moss AR, Osmond D, Bacchetti P, Torti FM, Gurgin V (1986). Hormonal risk-factors in testicular cancer—a case-control study. Am J Epidemiol.

[b63-ehp0116-000149] Mueller SO, Simon S, Chae K, Metzler M, Korach KS (2004). Phytoestrogens and their human metabolites show distinct agonistic and antagonistic properties on estrogen receptor alpha (ERalpha) and ERbeta in human cells. Toxicol Sci.

[b64-ehp0116-000149] National Center for Biotechnology Information (2007). PubMed.

[b65-ehp0116-000149] North K, Golding J (2000). A maternal vegetarian diet in pregnancy is associated with hypospadias. Br J Urol Int.

[b66-ehp0116-000149] Palmer JR, Wise LA, Robboy SJ, Titus-Ernstoff L, Noller KL, Herbst AL (2005). Hypospadias in sons of women exposed to diethylstilbestrol in utero. Epidemiology.

[b67-ehp0116-000149] Palmlund I, Apfel R, Buitendijk S, Cabau A, Forsberg JG (1993). Effects of diethylstilbestrol (des) medication during pregnancy—report from a Symposium at the 10th International-Congress of ISPOG. J Psychosomat Obstet Gynecol.

[b68-ehp0116-000149] Pierik FH, Burdorf A, Deddens JA, Juttmann RE, Weber RFA (2004). Maternal and paternal risk factors for cryptorchidism and hypospadias: a case-control study in newborn boys. Environ Health Perspect.

[b69-ehp0116-000149] Polednak AP, Janerich DT (1983). Maternal characteristics and hypospadias—a case-control study. Teratology.

[b70-ehp0116-000149] Pons JC, Papiernik E, Billon A, Hessabi M, Duyme M (2005). Hypospadias in sons of women exposed to diethylstilbestrol *in utero*. Prenat Diagn.

[b71-ehp0116-000149] Raman-Wilms L, Tseng AL, Wighardt S, Einarson TR, Koren G (1995). Fetal genital effects of first-trimester sex-hormone exposure—a meta-analysis. Obstet Gynecol.

[b72-ehp0116-000149] Restrepo M, Munoz N, Day N, Parra JE, Hernandez C, Blettner M (1990). Birth defects among children born to a population occupationally exposed to pesticides in Colombia. Scand J Work Environ Health.

[b73-ehp0116-000149] Richiardi L, Bellocco R, Adami HO, Torrang A, Barlow L, Hakulinen T (2004). Testicular cancer incidence in eight Northern European countries: secular and recent trends. Cancer Epidemiol Biomarkers Prev.

[b74-ehp0116-000149] Rothman KJ, Louik C (1978). Oral contraceptives and birth defects. N Engl J Med.

[b75-ehp0116-000149] Rushton L (2000). Reporting of occupational and environmental research: use and misuse of statistical and epidemiological methods. Occup Environ Med.

[b76-ehp0116-000149] Schottenfeld D, Warshauer ME, Sherlock S, Zauber AG, Leder M, Payne R (1980). The epidemiology of testicular cancer in young-adults. Am J Epidemiol.

[b77-ehp0116-000149] Shapiro E, Huang HY, Masch RJ, McFadden DE, Wu XR, Ostrer H (2005). Immunolocalization of androgen receptor and estrogen receptors alpha and beta in human fetal testis and epididymis. J Urol.

[b78-ehp0116-000149] Sharpe RM (2003). The ‘oestrogen hypothesis’—where do we stand now?. Int J Androl.

[b79-ehp0116-000149] Sharpe RM (2006). Pathways of endocrine disruption during male sexual differentiation and masculinisation. Best Pract Res Clin Endocrinol Metab.

[b80-ehp0116-000149] Sharpe RM, Skakkebaek NE (2003). Male reproductive disorders and the role of endocrine disruption: advances in understanding and identification of areas for future research. Pure Appl Chem.

[b81-ehp0116-000149] Skakkebaek NE, Rajpert-De Meyts E, Main KM (2001). Testicular dysgenesis syndrome: an increasingly common developmental disorder with environmental aspects. Hum Reprod.

[b82-ehp0116-000149] Sorensen HT, Pedersen L, Skriver MV, Norgaard M, Norgard B, Hatch EE (2005). Use of clomifene during early pregnancy and risk of hypospadias: population based case-control study. Br Med J.

[b83-ehp0116-000149] Starr JR, Chen C, Doody DR, Hsu L, Ricks S, Weiss NS (2005). Risk of Testicular germ cell cancer in relation to variation in maternal and offspring cytochrome p450 genes involved in catechol estrogen metabolism. Cancer Epidemiol Biomarkers Prev.

[b84-ehp0116-000149] Sterne JAC, Egger M, Smith GD, Egger M, Smith GD, Altman DG (2001). Investigating and dealing with publication and other biases. Systematic Reviews in Health Care: Meta Analysis in Context.

[b85-ehp0116-000149] Stoll C, Alembik Y, Roth MP, Dott B (1990). Genetic and environmental factors in hypospadias. J Med Genet.

[b86-ehp0116-000149] Storgaard L, Bonde JP, Olsen J (2006). Male reproductive disorders in humans and prenatal indicators of estrogen exposure—a review of published epidemiological studies. Reprod Toxicol.

[b87-ehp0116-000149] Strohsnitter WC, Noller KL, Hoover RN, Robboy SJ, Palmer JR, Titus-Ernstoff L (2001). Cancer risk in men exposed in utero to diethylstilbestrol. J Natl Cancer Inst.

[b88-ehp0116-000149] Sweet RA, Schrott HG, Kurland R, Culp OS (1974). Study of the incidence of hypospadias in Rochester, Minnesota, 1940–1970, and a case-control comparison of possible etiologic factors. Mayo Clin Proc.

[b89-ehp0116-000149] Toppari J, Larsen JC, Christiansen P, Giwercman A, Grandjean P, Guillette LJ (1996). Male reproductive health and environmental xenoestrogens. Environ Health Perspect.

[b90-ehp0116-000149] Torfs CP, Milkovich L, Van den Berg B (1981). The relationship between hormonal pregnancy tests and congenital anomalies: a prospective study. Am J Epidemiol.

[b91-ehp0116-000149] Veeramachaneni DNR (2000). Deteriorating trends in male reproduction: idiopathic or environmental?. Anim Reprod Sci.

[b92-ehp0116-000149] Vessey MP, Buckley J, Fairweather DV, Norman-Smith B, Buckley J (1983). A randomized double-blind controlled trial of the value of stilboestrol therapy in pregnancy: long-term follow-up of mothers and their offspring. Br J Obstet Gynaecol.

[b93-ehp0116-000149] Vidaeff AC, Sever LE (2005). In utero exposure to environmental estrogens and male reproductive health: a systematic review of biological and epidemiologic evidence. Reprod Toxicol.

[b94-ehp0116-000149] Vrijheid M, Armstrong B, Dolk H, van Tongeren M, Botting B (2003). Risk of hypospadias in relation to maternal occupational exposure to potential endocrine disrupting chemicals. Occup Environ Med.

[b95-ehp0116-000149] Walcott FL, Hauptmann M, Duphorne CM, Pillow PC, Strom SS, Sigurdson AJ (2002). A case-control study of dietary phytoestrogens and testicular cancer risk. Nutr Cancer.

[b96-ehp0116-000149] Watanabe M, Yoshida R, Ueoka K, Aoki K, Sasagawa I, Hasegawa T (2007). Haplotype analysis of the estrogen receptor 1 gene in male genital and reproductive abnormalities. Hum Reprod.

[b97-ehp0116-000149] Weidner IS, Moller H, Jensen TK, Skakkebaek NE (1998). Cryptorchidism and hypospadias in sons of gardeners and farmers. Environ Health Perspect.

[b98-ehp0116-000149] Weir HK, Marrett LD, Kreiger N, Darlington GA, Sugar L (2000). Prenatal and perinatal exposures and risk of testicular germ-cell cancer. Int J Cancer.

[b99-ehp0116-000149] Whitehead ED, Leiter E (1981). Genital abnormalities and abnormal semen analyses in male patients exposed to diethylstilbestrol in utero. J Urol.

[b100-ehp0116-000149] Yoshida R, Hasegawa T, Kamatani N, Fukami M, Sasagawa I, Ogata T (2005). Association of cryptorchidism with a specific haplotype of the estrogen receptor alpha gene: implication for the susceptibility to estrogenic environmental endocrine disruptors. J Clin Endocrinol Metabol.

[b101-ehp0116-000149] Zhang Y, Graubard BI, Klebanoff MA, Ronckers C, Stanczyk FZ, Longnecker MP (2005). Maternal hormone levels among populations at high and low risk of testicular germ cell cancer. Br J Cancer.

[b102-ehp0116-000149] Zhu Z, Boobis AR, Edwards RJ (2006). Use of protein profiles to characterise concentration-effect curves of mixtures of estrogenic compounds in human breast cell lines. Toxicol Lett.

